# Nobiletin Enhances Skeletal Muscle Mass and Modulates Bile Acid Composition in Diet-Induced Obese Mice

**DOI:** 10.1021/acs.jafc.5c00255

**Published:** 2025-04-07

**Authors:** Yen-Chun Koh, Chien-Ping Liu, Siu-Yi Leung, Wei-Sheng Lin, Pin-Yu Ho, Chi-Tang Ho, Min-Hsiung Pan

**Affiliations:** † Institute of Food Sciences and Technology, National Taiwan University, Taipei 10617, Taiwan; ‡ Department of Food Science, National Quemoy University, Quemoy 89250, Taiwan; § Department of Food Science, Rutgers University, New Brunswick, New Jersey 08901, United States; ∥ Department of Medical Research, China Medical University Hospital, China Medical University, Taichung City 40402, Taiwan

**Keywords:** obesity, muscle atrophy, glucose homeostasis, bile acid, gut microbiota

## Abstract

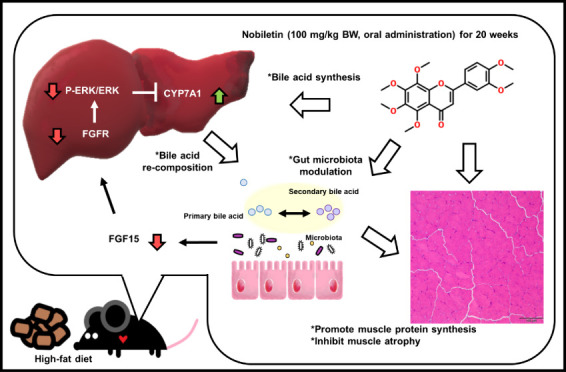

Obesity and its associated metabolic disorders—including muscle atrophy—pose significant health challenges, particularly with the increasing prevalence of high-fat diets. This study investigates the effects of nobiletin, a citrus flavonoid, on high-fat-diet-induced obesity-related muscle atrophy and its regulatory role in bile acid metabolism, aiming to determine whether nobiletin supplementation can enhance muscle mass and improve metabolic health in a mouse model. Our findings revealed that nobiletin significantly upregulated CYP7A1 expression in the liver, promoting bile acid synthesis and modulating bile acid composition in the ileum and feces, potentially through microbiota-mediated mechanisms. Furthermore, nobiletin supplementation suppressed muscle atrophy-related proteins, including p-4EBP1, TRIM63, and FBXO32, while promoting the phosphorylation of mTOR/AKT/p70S6K and FOXO3a in skeletal muscle. The FGF15/FGFR4/ERK signaling pathway was notably activated in the skeletal muscle tissues of nobiletin-supplemented mice, suggesting a protective effect against muscle atrophy despite the pathway’s inhibition in the liver to promote bile acid synthesis. These results indicate that nobiletin not only mitigates muscle atrophy in the context of obesity but also enhances glucose homeostasis, likely through improved skeletal muscle function. Overall, our study highlights the potential of nobiletin as a therapeutic agent for preventing obesity-related complications, regulating bile acid metabolism, and promoting skeletal muscle health.

## Introduction

The liver plays a crucial role in various metabolic and excretion processes, with cholesterol breakdown being a primary metabolic pathway for bile acid synthesis.^1^ Once formed, bile acids are secreted into the bile ducts and subsequently stored in the gallbladder. After food intake, the release of cholecystokinin from the small intestine stimulates gallbladder contraction, releasing bile acids into the gastrointestinal tract to aid in the emulsification, metabolism, and absorption of lipids. Most bile acids and nutrient-containing blood are absorbed at the ileal end through passive diffusion and active transport and then transported to the liver via the portal vein. This continuous recycling process is referred to as enterohepatic circulation.^2^ Bile acids serve as multifunctional signaling molecules that regulate endocrine functions throughout the body. They play a vital role in enterohepatic circulation by influencing nutrient absorption, distribution, and metabolic balance.^3^ Additionally, bile acids can activate signaling pathways involving FXR and TGR5, which help maintain homeostasis of triglycerides, cholesterol, energy, and glucose in the body. Consequently, conditions such as cholestasis, nonalcoholic fatty liver disease, and dysbiosis may arise when bile acid balance is disrupted and enterohepatic circulation is impaired.^4,5^

Previous studies indicate that gut microbiota dysbiosis can disrupt bile acid metabolism. For instance, antibiotic treatment in mice alters the structure and function of the gut microbiota, shifting bile acid composition from cholic acid (CA), taurocholic acid (TCA), and tauro-β-muricholic acid (T-β-MCA) to deoxycholic acid (DCA). DCA, a secondary bile acid, not only interacts with FXR to regulate bile acid synthesis and metabolism^6^ but is also associated with various liver diseases, liver cancer, and colorectal cancer.^7,8^ Other studies have shown that a high-fat diet (HFD) increases TCA levels, which, in turn, promotes the growth of the pathogenic bacterium *Bacteroides wadsworthia*. This alteration enhances proinflammatory immune responses and raises colitis incidence in mice.^9^ In pharmacological research, experiments using the antioxidant drug tempol in mice demonstrated a reduction in *Lactobacillus* populations and bile salt hydrolase activity, leading to increased T-β-MCA concentrations. Elevated T-β-MCA antagonizes the intestinal FXR/FGF19 signaling pathway, thereby stimulating bile acid synthesis and improving obesity and diabetes outcomes.^10^ Collectively, these findings suggest that dietary and pharmacological interventions can reshape the gut microbiota and bile acid profiles, potentially influencing gut health and liver disease progression through the FXR/FGF19 signaling pathway.

A HFD promotes fat and cholesterol accumulation in the liver, making it a major risk factor for nonalcoholic fatty liver disease (NAFLD) and cardiovascular diseases. This elevated risk may be due to increased passive bile acid absorption in the ileum under high-fat dietary conditions, which activates the ileal FXR-FGF15 signaling pathway. This activation suppresses CYP7A1 transcription in the liver, reducing cholesterol metabolism and leading to cholesterol accumulation.^11^ Studies indicate that HFDs also alter gut microbiota, disrupting bile acid balance and metabolism.^12^ For example, altered bile acid profiles have been observed in obese and nonalcoholic steatohepatitis (NASH) patients, characterized by increased conjugated bile acids and decreased secondary bile acids.^13^ Additionally, the relative abundance of Firmicutes and Bacteroidetes has been linked to obesity in both mice and humans, as these bacteria efficiently extract energy from HFDs, contributing to obesity. This dietary shift increases bile-resistant bacteria, decreases the Firmicutes-to-Bacteroidetes ratio, and raises TCA and DCA levels.^9^ These changes not only disrupt microbiota balance but also alter bile acid metabolism and bile acid pool size, ultimately influencing the progression of NASH and type 2 diabetes.

HFD-induced obesity in mice is associated with skeletal muscle atrophy, characterized by reduced muscle mass and fiber diameter, physical dysfunction, weakness, and metabolic impairments.^14^ Although bile acids are present at relatively low concentrations, they have been detected in muscle tissue.^15^ As gut microbiota-derived metabolites, bile acids can activate FXR, potentially influencing skeletal muscle physiology and playing a crucial role in obesity-induced sarcopenia.^16^ Given previous findings on nobiletin’s modulatory effects on gut microbiota^17^ and bile acid composition,^18^ this study explores its potential to mitigate HFD-induced muscle atrophy, particularly through bile acid regulation.

Nobiletin, a prominent polymethoxyflavone (PMF) found exclusively in citrus plants, particularly in the peel, contains six methoxy groups on its flavonoid backbone—positions 5, 6, 7, and 8 on the A-ring and 3′ and 4’ on the B-ring.^19^ Animal studies have demonstrated that nobiletin regulates lipid metabolism-related genes and reduces proinflammatory cytokine mRNA expression in HFD-induced C57BL/6J mice, alleviating obesity, dyslipidemia, hyperglycemia, and insulin resistance.^20^ In HFD-fed mice, nobiletin upregulates CPT1-α and PGC1-α, reducing hepatic lipid accumulation and VLDL-TG secretion while improving glucose tolerance, lowering hyperinsulinemia, and enhancing insulin sensitivity, suggesting its potential in preventing dyslipidemia and metabolic dysfunction.^21^ In 2024, nobiletin was shown to improve metabolism-associated fatty liver disease by regulating gut microbiota and bile acid metabolism.^18^ However, whether nobiletin’s modulation of bile acid metabolism can mitigate obesity-related muscle atrophy remains unclear.

## Materials and Methods

### Materials

Nobiletin (98% purity) was purchased from Nanjing Spring & Autumn Biological Engineering (Nanjing, China). Antibodies used in this study were sourced as follows: anti-FXR (NR1H4), FOXO3a, FGFR4, Klotho, SIRT1, PGC-1α, Vinculin, and GAPDH from Proteintech (Rosemont, IL, USA); anti-p-Akt, t-Akt, p-ERK, ERK, p-mTOR, t-mTOR, and p-FOXO3a from Cell Signaling Technology (Danvers, MA, USA); and p-Eukaryotic translation initiation factor 4E (eIF4E)-binding protein 1 (4EBP1), F-box only protein 32 (FBXO32), p-Ribosomal protein S6 kinase beta-1 (p70S6K), t-p70S6K, and Muscle Ring-Finger Protein-1 (TRIM63), MyoD1, and myogenin from ABclonal Biotech Co., Ltd. (China). FGF15 and CYP7A1 antibodies were obtained from Santa Cruz Biotechnology (Dallas, TX, USA), while anti-β-actin was purchased from Sigma-Aldrich (MA, USA).

### In Vitro Study

The in vitro study was conducted using the C2C12 murine myoblast cell line (ATCC). Cells were cultured in DMEM supplemented with 10% heat-inactivated FBS (Gibco). Initially, they were seeded at a density of 1 × 10^4^ cells per well and incubated for 12 h. To induce differentiation, the culture medium was replaced with DMEM containing 5% horse serum, with medium changes every 2 days. Cell morphology was monitored through microscopy, and differentiation was completed by day 6. Dexamethasone (DEX, 1 μM), CA (500 μM), or DCA (120 μM) were used as inducers.

### Animal Study

A mouse model was used in this experiment. Four-week-old male C57BL/6J mice were acclimated for 1 week, during which no significant differences in average body weight were observed between groups. The mice were then randomly assigned to four groups (*n* = 8 per group): (1) Normal diet (ND), receiving a standard chow diet (Laboratory Rodent Diet 5001); (2) HFD, fed Research Diet D12492 (60 kcal% fat); (3) low-dose nobiletin (LNB), receiving 20 mg/kg body weight (BW) nobiletin via oral gavage alongside the HFD; and (4) high-dose nobiletin (HNB), receiving 100 mg/kg BW nobiletin on the HFD. Nobiletin was suspended in 0.5% carboxymethyl cellulose (CMC), while the ND and HFD groups received only 0.5% CMC suspension via oral gavage. All groups were provided pellet feed on cage racks for ad libitum feeding and had free access to water. The experiment lasted 20 weeks, after which mice were sacrificed. Blood, organs, and feces were collected; organs were photographed, weighed, and stored at −80 °C.

### Dosage Selection

A previous study found that administering nobiletin (17 mg/kg BW/day) to mice for 16 weeks, without changes in food intake or body weight, improved glucose tolerance, insulin resistance, and reduced hypercholesterolemia induced by a HFD.^22^ Therefore, in this experiment, a dose of nobiletin (20 mg/kg BW/day) was selected for the low-dose group. Additionally, another study demonstrated that administering a high dose of nobiletin (100 mg/kg BW/day) to mice fed a HFD not only reduced average food intake but also improved gut microbiota composition and increased the formation of short-chain fatty acids. Moreover, fecal microbiota transplantation experiments confirmed that the nobiletin-altered gut microbiota could confer health benefits and antiobesity potential in mice.^17^ Consequently, in this experiment, a dose of nobiletin (100 mg/kg BW/day) was chosen for the high-dose group.

### Biochemical Parameter Analysis

Blood was collected via heart puncture and centrifuged at 4 °C, 4200×*g* for 10 min. The resulting supernatant was transferred to a separate container as serum, which was stored at −80 °C. Serum samples were later sent to the National Laboratory Animal Center for biochemical analysis, including aspartate aminotransferase (AST), alanine aminotransferase (ALT), glucose (GLU), total cholesterol (T-CHO), triglycerides (TG), high-density lipoprotein cholesterol (HDL-C), and low-density lipoprotein cholesterol (LDL-C).

### Fasting Glucose, Fasting Insulin, and the Oral Glucose Tolerance Test (OGTT)

The method for measuring fasting blood glucose involved an 8 h fasting period for the mice. During fasting, food was removed, bedding was replaced, and water bottles were retained in the cages. After the fasting period, approximately 0.5 mm of the tail tip was cut using the tail snip method to collect a small blood sample for fasting glucose measurement. Following this, the mice were orally administered glucose at a dosage of 2 g/kg body weight, and blood glucose levels were measured at 30, 60, 90, and 120 min postadministration. Fasting insulin levels were measured using the Mercodia Mouse Insulin ELISA kit (Cat. 10-1247-01, Sweden), following the manufacturer’s protocol. The HOMA-IR (Homeostatic Model Assessment for Insulin Resistance) was calculated using the following equation:
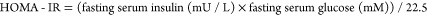


### Hepatic and Muscle Triglyceride Content

The triglyceride content in the liver and gastrocnemius muscle tissues was measured using a triglyceride colorimetric assay kit (Cayman, 10010303) according to the manufacturer’s protocol. The tissues were weighed and homogenized, and the supernatants were subsequently collected for analysis.

### Hematoxylin and Eosin (H&E) Staining Procedure

For histopathological analysis, liver and gastrocnemius muscle tissues were subjected to H&E staining to visualize structural details. The harvested tissue samples were initially fixed in 10% formalin buffer. After fixation, the tissues were dehydrated, embedded in paraffin, and sectioned into 3–5 μm thick slices. The sections were then deparaffinized with xylene and rehydrated through a graded ethanol series before undergoing H&E staining.

### Western Blot

Liver, gastrocnemius muscle tissue, and ileal proteins were homogenized and lysed using ice-cold lysis buffer, followed by at least 1 h of incubation on ice. After homogenization, the samples were centrifuged at 14,000*g* for 1 h at 4 °C. The supernatants were collected and stored at −80 °C for further analysis. Protein concentrations were determined using the Bio-Rad protein assay. For electrophoresis, 25 μg of protein was loaded into each well and transferred onto PVDF membranes (Merck Millipore Ltd., Tullagreen, County Cork, Ireland). The membranes were then blocked using a solution containing 20 mM Tris-base, 137 mM NaCl, 1% BSA (w/v), 1% Tween 20, and 0.1% sodium azide, followed by overnight incubation with primary antibodies. The membranes were washed multiple times with 0.2% TPBS (phosphate-buffered saline with Tween 20) to ensure proper antibody binding and removal of unbound antibodies, both before and after the application of secondary antibodies. Protein bands were visualized using chemiluminescence (ECL, Merck Millipore Ltd.), and densitometric analysis was performed using ImageJ software. GAPDH, β-actin, and vinculin were used as internal controls for the Western blotting.

### Bile Acid Content Analysis

For bile acid analysis, approximately 100 mg of cecal and ileal contents were collected from each mouse and stored individually in 2 mL Eppendorf tubes at −80 °C. The samples were then freeze-dried using a lyophilizer, and 25 mg of the freeze-dried material was transferred to a separate microtube for further analysis. Bile acid quantification was performed by the NTU Mass Spectrometry Consortia of Key Technologies at National Taiwan University using the SCIEX Triple Quad 5500+ LC–MS/MS system. The following bile acids were measured: CA, chenodeoxycholic acid (CDCA), DCA, lithocholic acid (LCA), ursodeoxycholic acid (UDCA), taurochenodeoxycholic acid (TCDCA), tauroursodeoxycholic acid (TUDCA), β-muricholic acid (β-MCA), α-muricholic acid (α-MCA), TCA, and T-β-MCA.

### Gut Microbiota Analysis

Using the stool DNA extraction kit (Analytik Jena GmbH, innuPREP Stool DNA Kit), fecal samples underwent lysis and filtration to remove insoluble materials. The resulting filtrate was passed through a membrane to capture DNA, which was then eluted through repeated centrifugation and washing steps for further applications. For full-length 16S ribosomal gene analysis, PCR was conducted using labeled universal primers (27F + 1492R) to amplify the 16S gene from the samples. The labeled DNA underwent quality control and was mixed for SMRTbell library preparation, followed by purification with AMPure PB Beads, DNA repair, and adapter ligation before sequencing. Bioinformatics analysis utilized the sequencing results, beginning with quality control of the raw sequences (Polymerase Read) and overlapping subreads. Only those with more than three overlaps were used to generate consensus sequences. High-quality and long sequencing reads (RQ > 20) were classified as HiFi reads. For improved species resolution, higher-quality HiFi reads (RQ > 30) were employed for comprehensive microbial 16S ribosomal gene analysis, achieving a base accuracy of 99.9%. The HiFi sequences underwent DADA2 analysis to produce Amplicon Sequence Variants (ASVs) through quality control, dereplication, chimera removal, and sequence aggregation. The generated ASVs were then compared with databases (NCBI, GreenGenes, SILVA, eHOMD, and UNITE) to obtain species information, resulting in a species abundance table.

## Results

### Dietary Intake of Nobiletin Significantly Prevents Body Weight Gain Induced by a High-Fat Diet

**Figure 1:**
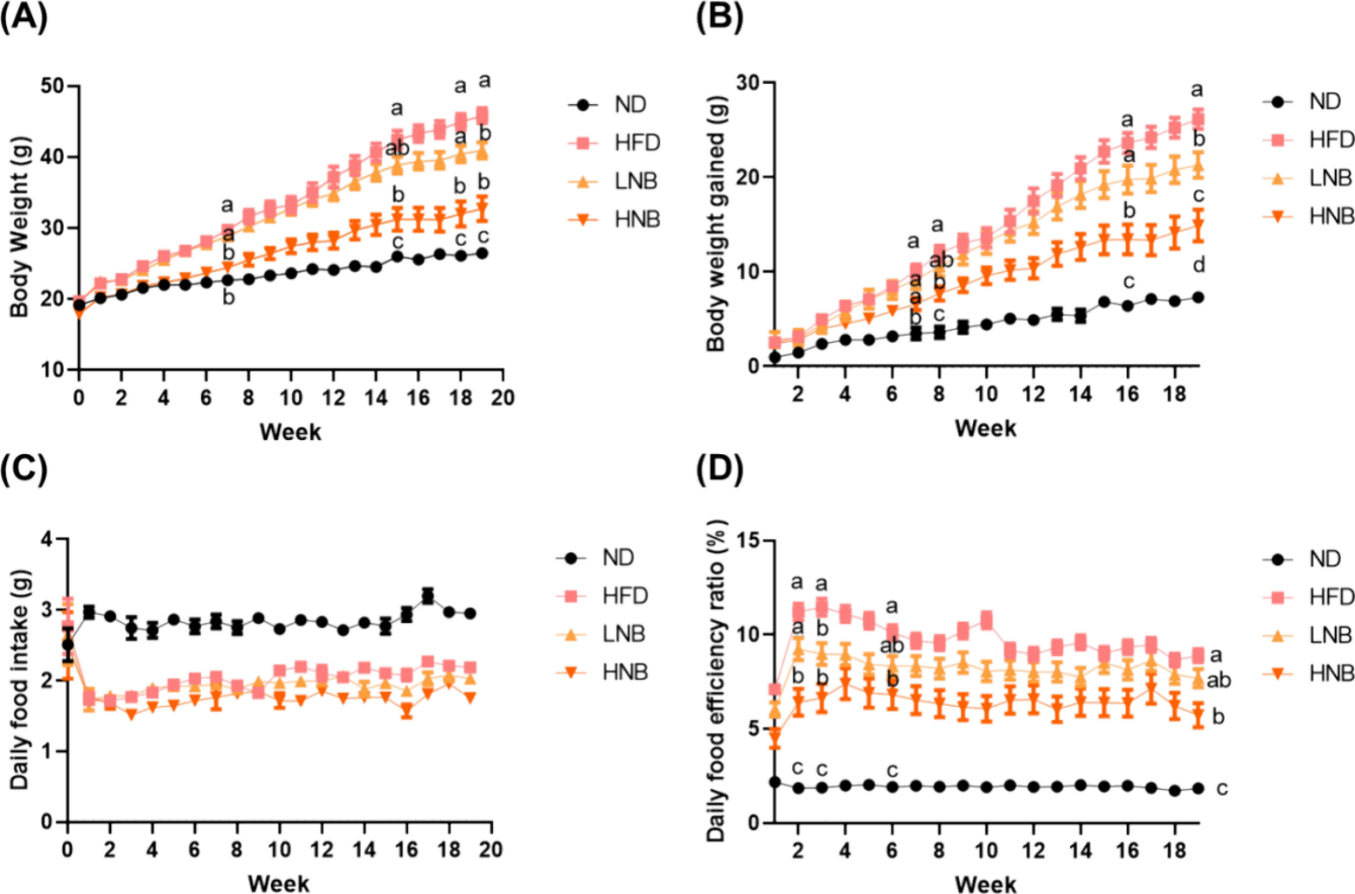
Nobiletin supplementation significantly prevents body weight gain induced by a high-fat diet. (A) Changes in body weight and (B) body weight gain after 20 weeks of feeding with a high-fat diet and nobiletin supplementation. (C) Daily food intake and (D) daily food efficiency ratio (%) are shown. The food efficiency ratio is calculated as body weight gain (g)/food intake (g) × 100. All data are presented as means ± S.E., *N* = 8. Different lowercase letters indicate significant differences among groups, as determined by ANOVA followed by Tukey’s post hoc test.

After 19 weeks on a HFD, mice exhibited significant weight gain, with statistical differences emerging as early as week 7. High-dose nobiletin supplementation effectively prevented weight gain without altering food or water intake, whereas low-dose supplementation had no significant effect (Figure 1A). Significant differences in body weight and weight gain were observed between the ND and HNB groups, indicating that while high-dose nobiletin mitigated some adverse effects of the HFD, it did not fully prevent them (Figure 1A,B). By week 19, both low- and high-dose nobiletin supplementation showed beneficial effects in reducing weight gain, with a dose-dependent reduction observed in the final week before sacrifice. Additionally, the food efficiency ratio, which measures the relationship between food intake and body weight gain, was lower in the high-dose nobiletin group. This reduction was independent of food intake, suggesting that nobiletin may reduce the conversion of ingested food into body mass (Figure 1D).

### Nobiletin Supplementation Reverses the Detrimental Effects of a High-Fat Diet on Organ Health

**Figure 2:**
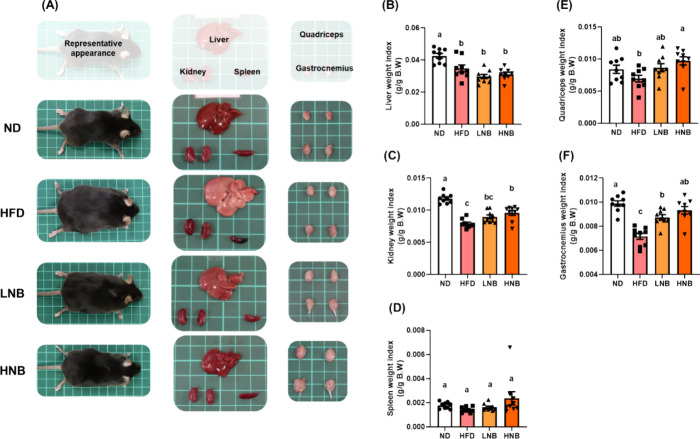
Representative images of mouse appearances and organ characteristics. (A) Representative images showing the overall appearance of the mice, along with liver, kidney, spleen, quadriceps, and gastrocnemius skeletal muscle tissues. The organ weight indices for (B) liver, (C) kidney, (D) spleen, (E) quadriceps, and (F) gastrocnemius muscle are presented. All data are expressed as means ± S.E., with *N* = 8. Different lowercase letters indicate significant differences among groups, as determined by ANOVA followed by Tukey’s post hoc test.

**Figure 3:**
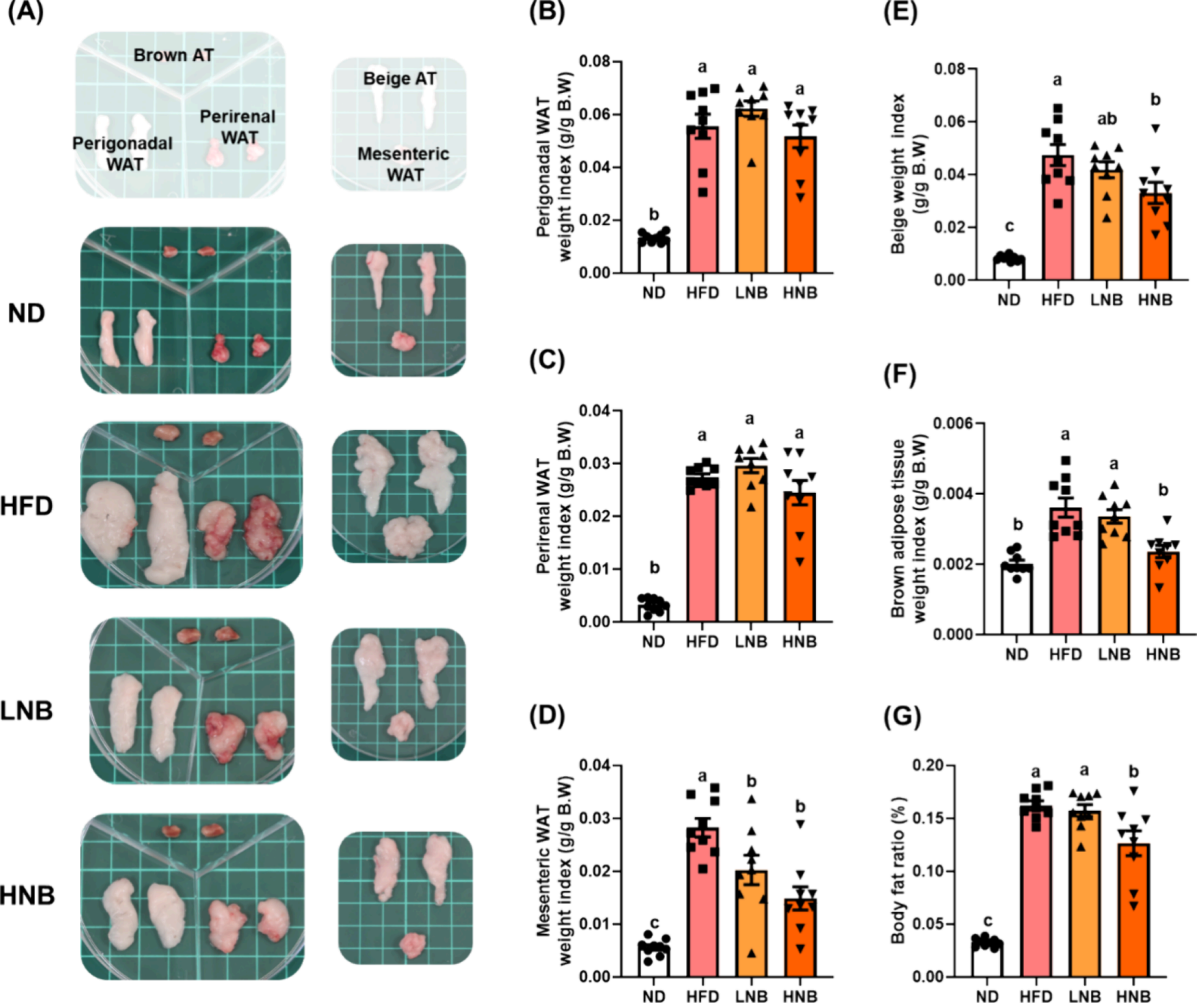
Nobiletin supplementation alleviates the adverse effects of a high-fat diet on adipose tissue. (A) Representative images of various adipose tissues, including brown, beige, mesenteric, perigonadal, and perirenal adipose tissues. The weight indices for (B) perigonadal white adipose tissue, (C) perirenal white adipose tissue, (D) mesenteric white adipose tissue, (E) beige adipose tissue, (F) brown adipose tissue, and (G) body fat ratio (%) are presented. The body fat ratio is calculated by summing the weights of all adipose tissues, dividing by the body weight, and multiplying by 100%. All data are expressed as means ± S.E., with *N* = 8. Different lowercase letters indicate significant differences among groups, as determined by ANOVA followed by Tukey’s post hoc test.

Feeding the mice a HFD for 19 weeks resulted in several noticeable changes in physical appearance. As shown in the representative images in Figure 2, the body shape of the HFD group displayed the most pronounced differences compared to the ND group, with the LNB and HNB groups showing intermediate changes. Observable alterations in organ appearance included a larger, paler liver in the HFD group and the enlargement of various adipose tissues, such as perigonadal, perirenal, mesenteric, beige, and brown adipose tissues. Figure 2 also presents the organ weight indices from this study. A significant reduction in liver and kidney weight indices was observed in the HFD group, with high-dose nobiletin reversing the reduction in kidney weight index (Figure 2C). However, when organ weight was normalized to body weight, these changes were less pronounced in the weight indices of obese mice. As shown in Figure S1G, liver weight significantly increased in the HFD group, a change that was effectively prevented by both low-dose and high-dose nobiletin supplementation. Notably, the quadriceps and gastrocnemius muscle weights were significantly lower in the HFD group compared to those in the nobiletin-supplemented groups (Figure 2E,F). The weight indices of various adipose tissues were significantly higher in the HFD group, but high-dose nobiletin (HNB group) supplementation effectively reduced the weight of mesenteric, beige, and brown adipose tissues, as well as the overall body fat ratio (Figure 3).

### Nobiletin Prevents the High-Fat Diet-Induced Elevation of Serum Total Cholesterol and Triglyceride Levels

H&E staining of liver sections was performed to confirm the beneficial effects of nobiletin on lipid accumulation (Figure S2). In the HFD group, macrovesicular fat, immune cell infiltration, and hepatocyte ballooning were observed, which contributed to the increased liver weight (Figure S1K). Low-dose nobiletin partially alleviated lipid accumulation, while high-dose nobiletin effectively prevented the adverse effects induced by the HFD (Figure S2). These findings were further supported by the liver TG content data shown in Table 1, where nobiletin supplementation significantly reduced hepatic TG levels.

**Table 1:** Biochemical Parameters and Glucose Homeostasis-Related Indicators^a^

	ND	HFD	LNB	HNB
AST (U/L)	248.54 ± 176.37^a^	265.58 ± 79.34^a^	230.23 ± 79.88^a^	256.14 ± 100.82^a^
ALT (U/L)	32.36 ± 7.92^a^	72.28 ± 40.49^a^	67.01 ± 68.20^a^	28.01 ± 14.51^a^
GLU (mg/dL)	288.34 ± 74.29^b^	438.33 ± 57.13^a^	362.48 ± 72.21^ab^	316.32 ± 103.59^b^
T-CHO (mg/dL)	84.56 ± 8.95^c^	224.01 ± 15.36^a^	193.18 ± 42.85^ab^	180.06 ± 43.23^b^
TG (mg/dL)	84.38 ± 51.89^a^	86.03 ± 28.08^a^	62.70 ± 27.71^a^	55.60 ± 18.04^a^
HDL (mg/dL)	70.06 ± 6.95^b^	169.54 ± 9.94^a^	152.93 ± 33.57^a^	141.03 ± 38.23^a^
LDL (mg/dL)	9.16 ± 0.84^b^	35.08 ± 3.79^a^	31.26 ± 6.81^a^	30.73 ± 4.95^a^
LDL/HDL ratio	0.13 ± 0.02^b^	0.21 ± 0.01^a^	0.21 ± 0.02^a^	0.24 ± 0.1^a^
HDL/T-CHO ratio	0.83 ± 0.04^a^	0.76 ± 0.03^b^	0.79 ± 0.03^ab^	0.77 ± 0.06^b^
LDL/T-CHO ratio	0.11 ± 0.02^b^	0.16 ± 0.01^a^	0.16 ± 0.01^a^	0.18 ± 0.05^a^
liver TG (mg/g protein)	165.62 ± 82.42^c^	706.82 ± 128.55^a^	453.13 ± 164.36^b^	379.82 ± 118.72^b^
fasting insulin	0.44 ± 0.07^b^	2.89 ± 1.41^a^	1.30 ± 0.48^ab^	0.85 ± 0.29^b^
HOMA-IR	6.03 ± 1.27^b^	74.66 ± 35.87^a^	28.88 ± 18.56^ab^	14.60 ± 8.22^b^
AUC	20076 ± 1116^b^	28551 ± 3813^a^	24253 ± 2292^ab^	22512 ± 2285^b^

a Different lowercase letters indicate significant differences among groups, as determined by ANOVA followed by Tukey’s post hoc test.

The biochemical parameters are presented in Table 1. The HFD group exhibited an increasing trend in serum AST and ALT levels, which were reduced by nobiletin supplementation, although the reduction did not reach statistical significance. Additionally, feeding with a HFD significantly elevated serum T-CHO, TG, fasting glucose, HDL, and LDL levels. Nobiletin supplementation effectively ameliorated fasting glucose, T-CHO, and TG levels, with the most pronounced effects observed at the high dosage. Similar improvements were also noted in fasting insulin, HOMA-IR, and OGTT, highlighting the potential of high-dose nobiletin in improving glucose homeostasis.

### Nobiletin Prevents Obesity-Related Sarcopenia, Possibly via Activation of the mTOR/Akt Pathway

As shown in Figure S2, muscle fibers were tightly packed in the ND group, whereas the interstitial space increased in the HFD group. The muscle fibers also appeared disorganized, with some immune cell infiltration observed in the HFD group. These changes were ameliorated in the LNB group and, more significantly, in the HNB group.

**Figure 4:**
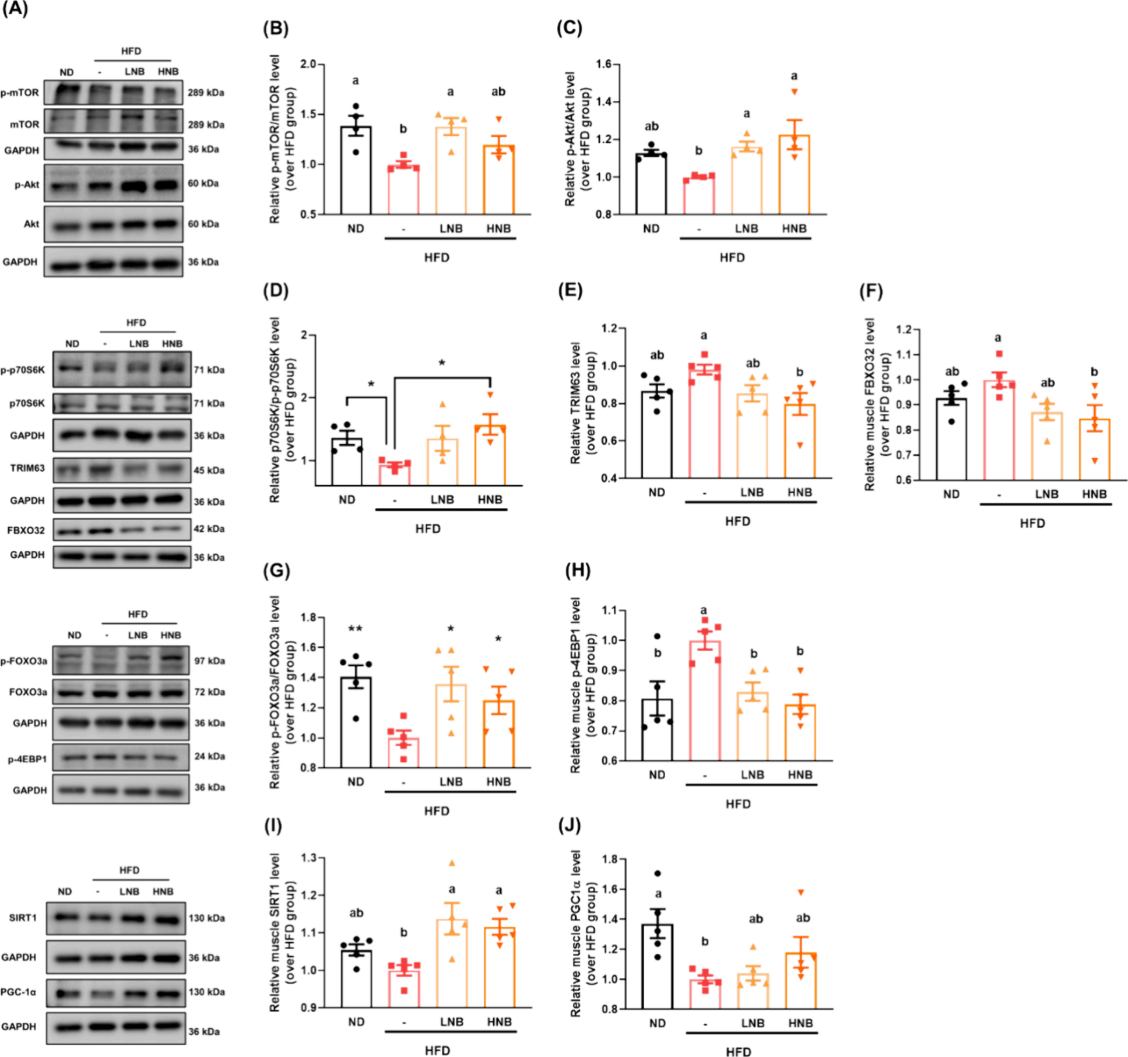
Nobiletin mitigates obesity-related sarcopenia, potentially through activation of the mTOR/Akt pathway. (A) Representative images of Western blot analysis and quantification of protein expression for (B) p-mTOR/mTOR, (C) p-Akt/Akt, (D) p-p70S6K/p70S6K, (E) TRIM63, (F) FBXO32, (G) p-FOXO3a/FOXO3a, (H) p-4EBP1, (I) SIRT1, and (J) PGC-1α in gastrocnemius muscle tissues. GAPDH was used as the internal control. All data are presented as means ± SE. At least 4 independent biological replicates were included for the Western blot analysis. Different lowercase letters indicate significant differences among groups, as determined by ANOVA followed by Tukey’s post hoc test.

Western blot analysis was performed to explore the underlying mechanisms through which nobiletin prevents muscle weight loss. Our results showed that the HFD significantly reduced the p-mTOR/mTOR and p-Akt/Akt ratios, while nobiletin supplementation markedly increased the phosphorylation of both mTOR and Akt. Additionally, nobiletin promoted the phosphorylation of p70S6K (Figure 4D), which enhances protein synthesis, as well as FOXO3a (Figure 4F), a key protein that inhibits protein degradation. The protein degradation-related marker TRIM63, a downstream target of FOXO3a, was significantly downregulated by nobiletin, suggesting that nobiletin supplementation effectively prevents HFD-induced muscle protein degradation.

**Figure 5:**
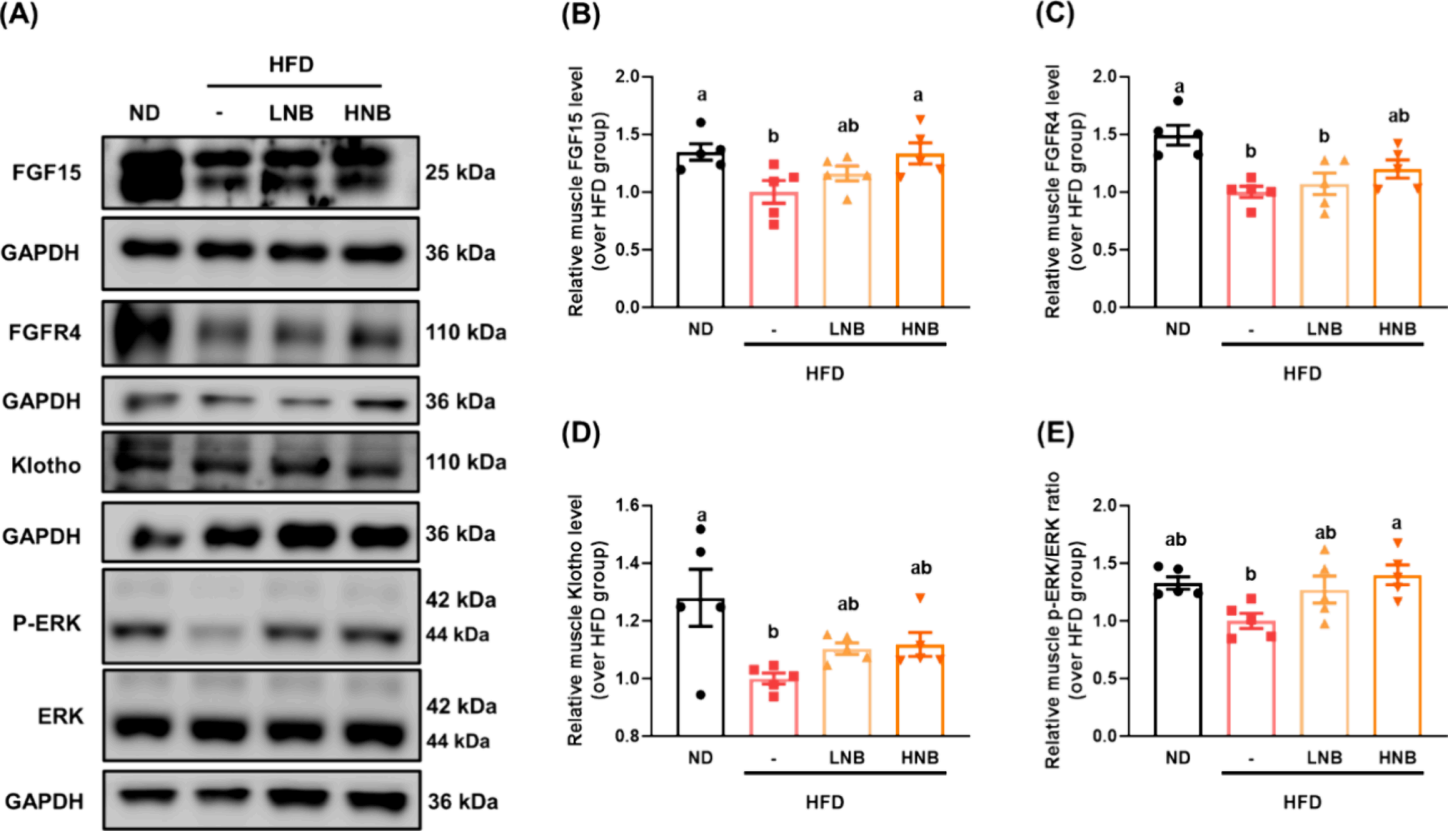
Activation of the FGF15/FGFR4/ERK signaling pathway may contribute to enhanced muscle synthesis. (A) Representative images of Western blot analysis and quantification of protein expression for (B) FGF15, (C) FGFR4, (D) Klotho, and (E) p-ERK/ERK in gastrocnemius muscle tissues. GAPDH was used as the internal control. All data are presented as means ± SE. At least 4 independent biological replicates were included in the Western blot analysis. Different lowercase letters indicate significant differences among groups, as determined by ANOVA followed by Tukey’s post hoc test.

To confirm the effect of nobiletin against muscle atrophy (Figure 5), we conducted in vitro studies. The results showed that nobiletin could not reverse the upregulation of atrophy markers FBXO32 and TRIM63 induced by dexamethasone, suggesting that its muscle-enhancing effects may occur through an indirect mechanism (Figure S4). However, nobiletin promoted MyoD1 expression and reversed the downregulation of myogenin caused by cholic acid (Figure S5). Therefore, we further analyzed bile acid metabolism and composition in the following sections.

### Nobiletin Supplementation Regulates Hepatic Bile Acid Synthesis

**Figure 6:**
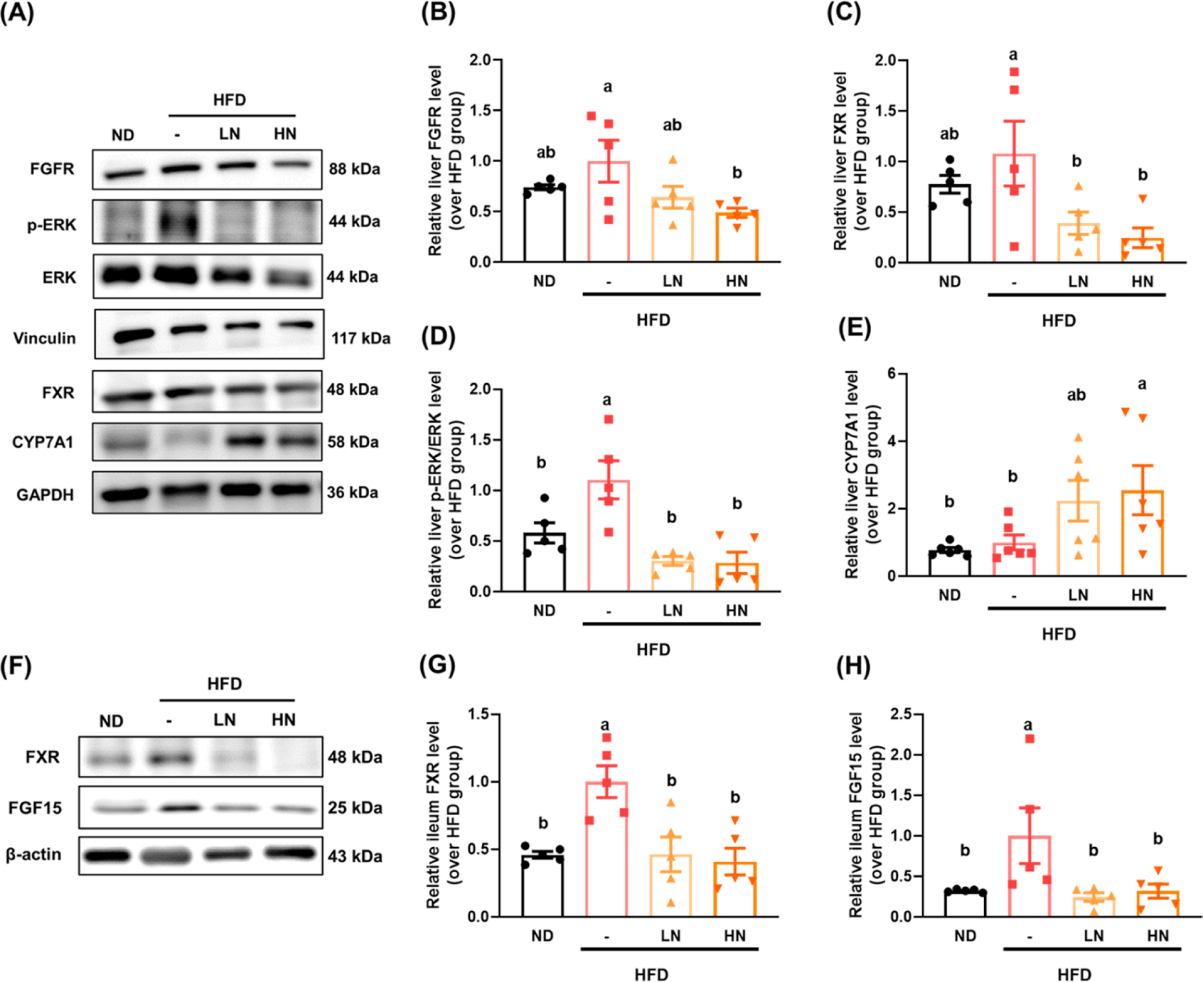
Inhibition of the FGFR/FXR/ERK signaling pathway by nobiletin supplementation promotes bile acid synthesis via CYP7A1. (A) Representative images of Western blot analysis and quantification of protein expression for (B) FGFR4, (C) FXR, (D) p-ERK/ERK, and (E) CYP7A1 in liver tissues. (F) Representative images of Western blot analysis and quantification of protein expression for (G) FXR and (H) FGF15 in the ileum tissue. Vinculin, GAPDH, and β-actin were used as internal controls. All data are presented as means ± SE. At least 4 independent biological replicates were included in the Western blot analysis. Different lowercase letters indicate significant differences among groups, as determined by ANOVA followed by Tukey’s post hoc test.

As mentioned earlier, serum cholesterol levels were significantly elevated in the HFD group, and this dysregulation was prevented by nobiletin supplementation (Table 1). Both de novo cholesterol synthesis and dietary cholesterol uptake contribute to hepatic bile acid synthesis through the classic and alternative pathways. Our results showed that a HFD significantly upregulated hepatic FGFR (Figure 6B) and FXR (Figure 6C) expression, leading to a subsequent reduction in the expression of hepatic CYP7A1 (Figure 6E), the key enzyme responsible for converting cholesterol into bile acids via the classic pathway. The upregulation of phosphorylated ERK (Figure 6D) further supported the inhibition of CYP7A1, resulting in reduced bile acid synthesis. In contrast, ileal FXR and FGF15 levels were significantly elevated in the HFD group; however, nobiletin supplementation markedly reduced their expression (Figure 6F–H). Since the activation of FXR and FGF15 is regulated by bile acids, the composition of bile acids was analyzed in the subsequent section.

### Regulatory Effect of Nobiletin on Ileal and Fecal Bile Acid Composition

**Figure 7:**
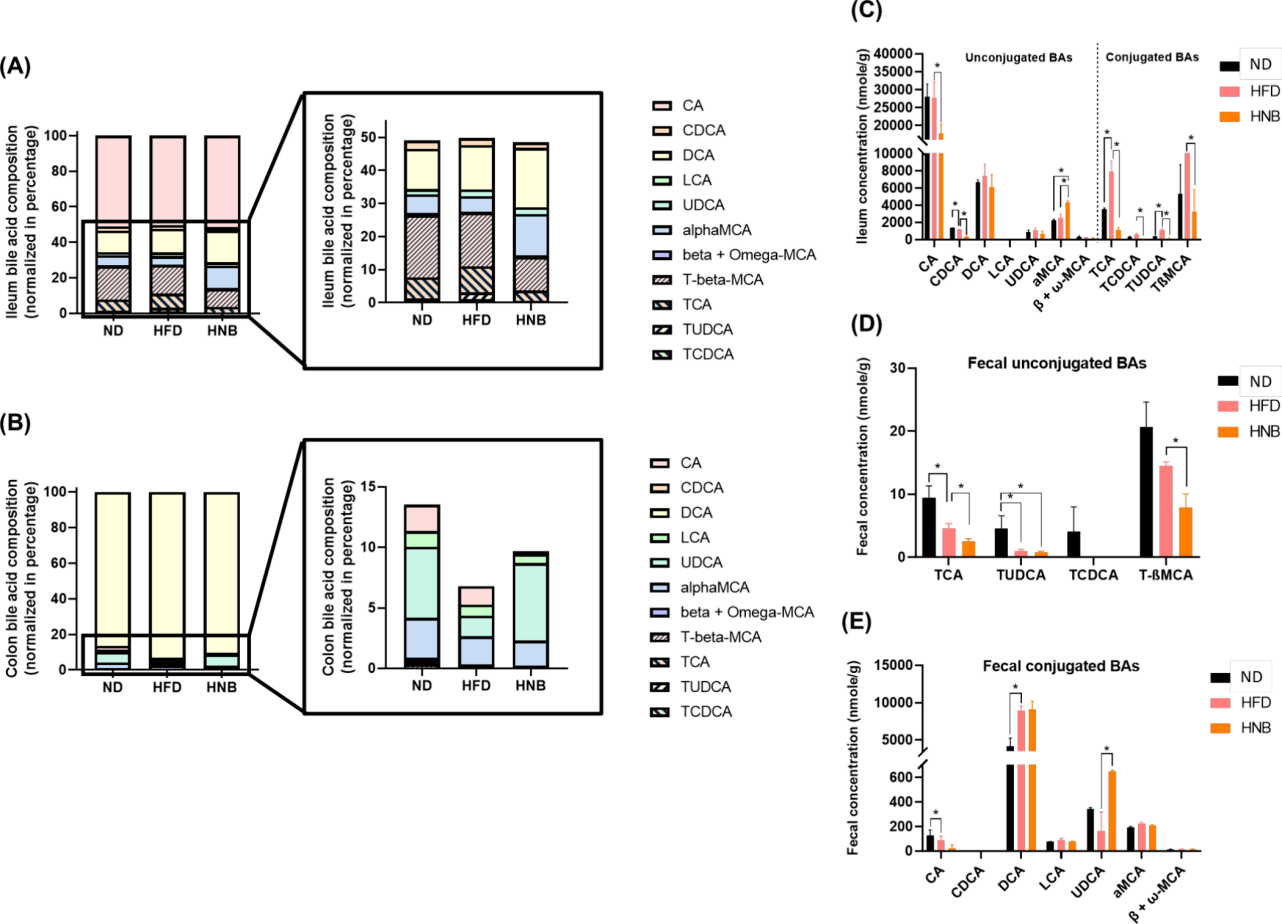
Changes in bile acid composition and concentration following HFD feeding and nobiletin intervention. (A) Ileal and (B) colonic fecal bile acid composition (normalized as a percentage). (C) Ileal and (D,E) colonic fecal bile acid concentration. All data are presented as means ± SE (*N* = 4). (*) Indicates a significant difference between compared groups, as determined by Student’s *t* tests. CA, cholic acid; CDCA, chenodeoxycholic acid; DCA, deoxycholic acid; LCA, lithocholic acid; UDCA, ursodeoxycholic acid; α-MCA, alpha-muricholic acid; β-MCA, beta-muricholic acid; TCA, taurocholic acid; TUDCA, tauroursodeoxycholic acid; TCDCA, taurochenodeoxycholic acid; TβMCA, tauro-beta-muricholic acid.

The bile acid compositions (normalized as percentages) are presented in Figure 7A,B, while the concentrations are shown in Figure 7C,D. In terms of composition, the levels of DCA and alpha-MCA were comparatively higher in the ileum of the HNB group than in the HFD group (Figure 7A). Additionally, high-dose nobiletin supplementation reduced ileal levels of CDCA, T-beta-MCA, TCA, TUDCA, and TCDCA (Figure 7A), as well as fecal CA levels (Figure 7B). Notably, the percentage of UDCA was significantly higher in both the ND and HNB groups compared to the HFD group (Figure 7B).

A comparison of bile acid concentrations revealed that the HFD significantly elevated ileal TCA and TUDCA levels (Figure 7C), increased fecal DCA, and reduced fecal CA levels (Figure 7D,E). However, nobiletin supplementation notably reduced ileal CA, CDCA, TCA, TCDCA, TUDCA, and T-beta-MCA levels (Figure 7C), as well as fecal TCA and T-beta-MCA levels (Figure 77D). Additionally, nobiletin supplementation significantly increased ileal alpha-MCA (Figure 7C) and fecal UDCA (Figure 7E) levels. Since changes in bile acid composition are closely linked to the host gut microbiota, these results prompted an analysis of gut microbiota composition.

### Modulatory Effect of Nobiletin on High-Fat Diet-Induced Gut Microbial Dysbiosis

**Figure 8:**
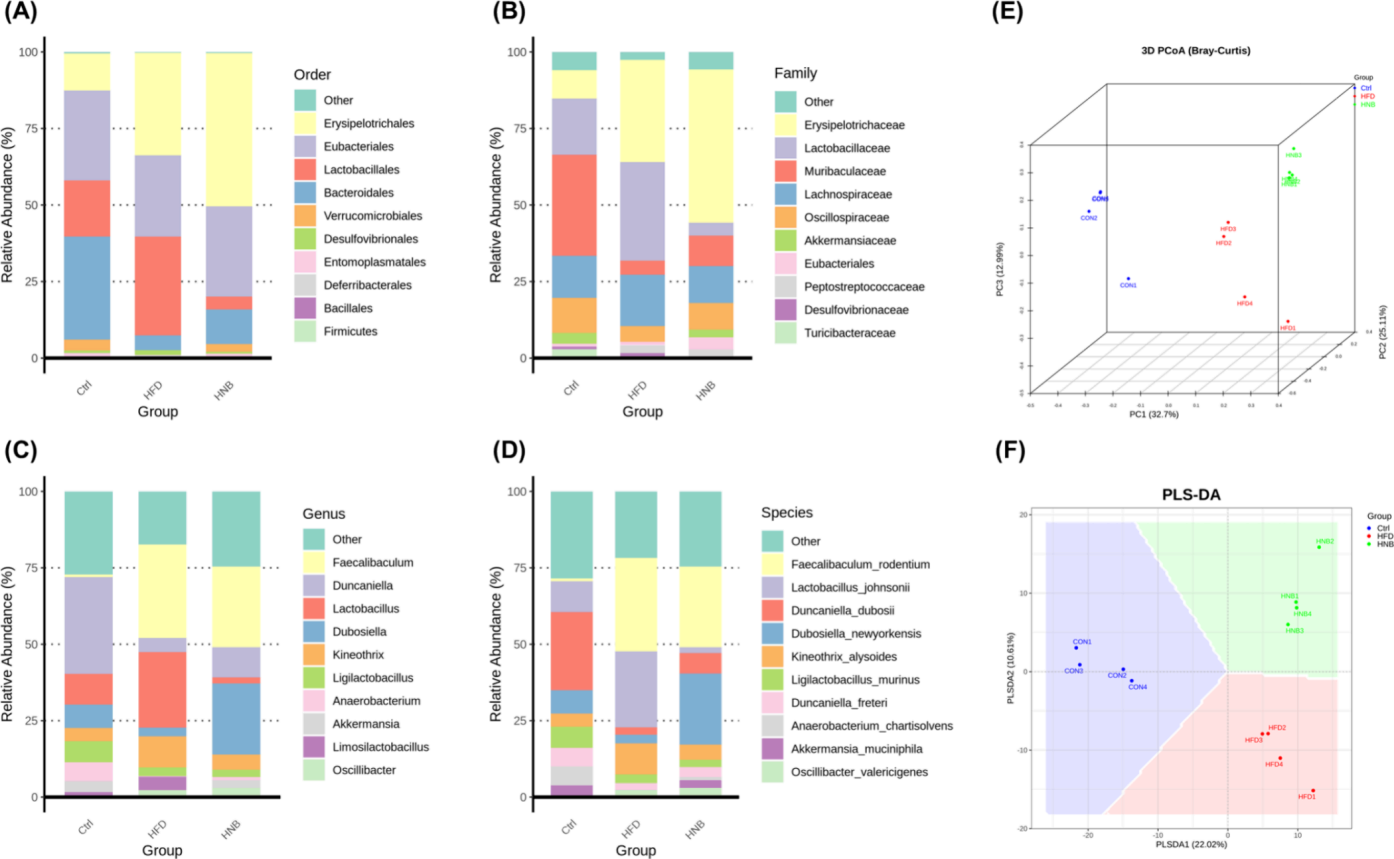
Changes in gut microbiota composition following high-fat diet feeding and nobiletin intervention. The top ten most abundant gut microbes at the taxonomic rank of (A) order, (B) family, (C) genus, and (D) species. (E) 3D principal coordinates analysis (PCoA) based on Bray–Curtis dissimilarity. (F) Partial least-squares discriminant analysis (PLS-DA) plot.

The top 10 most abundant gut microbes at the taxonomic ranks of order, family, genus, and species are shown in Figure 8A–D, respectively. There were substantial shifts in the gut microbial composition of the HFD group compared to the ND group, while high-dose nobiletin supplementation exhibited a notable modulatory effect on the restructuring of the gut microbiota. The results of principal coordinates analysis and partial least-squares discriminant analysis further indicated that the HFD significantly altered the gut microbiota composition, and high-dose nobiletin supplementation effectively reshaped the host’s gut microbial community.

**Figure 9:**
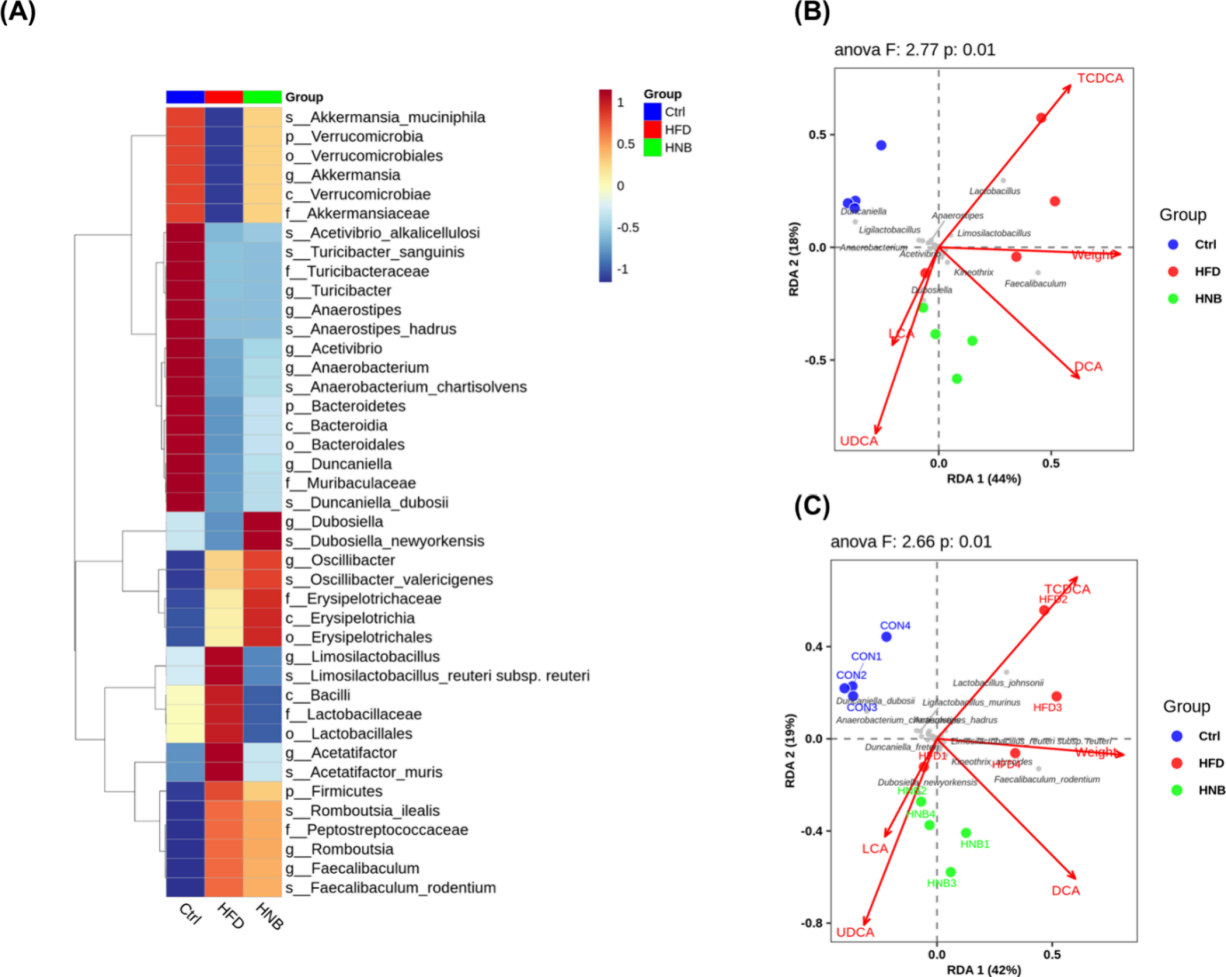
Gut microbial biomarkers for each group and RDA correlation plot between gut microbes and environmental factors. (A) Linear discriminant analysis (LDA) effect size (LEfSe) with an LDA score threshold of 3.0. (B) Redundancy analysis (RDA) plot showing gut microbes at the genus level. (C) RDA plot showing gut microbes at the species level.

Figure 9A shows the results of the linear discriminant analysis (LDA) effect size analysis at an LDA score of 3.0. Our findings revealed that the HFD drastically reduced the abundance of *Akkermansia muciniphila* while increasing *Limosilactobacillus reuteri*, *Acetatifactor muris*, *Romboutsia ilealis*, and *Faecalibaculum rodentium*. These changes were reversed by high-dose nobiletin supplementation. However, the HFD-induced reduction in *Acetivibrio alkalicellulosi*, *Turicibacter sanguinis*, and *Anaerostipes hadrus* was not reversed by the treatment. Meanwhile, the decrease in *Anaerobacterium chartisolvens* and *Duncaniella dubosii* was partially restored by nobiletin. Notably, nobiletin also promoted the growth of *Dubosiella newyorkensis* and *Oscillibacter valericigenes*.

According to the RDA (redundancy analysis) plot, the increase in *Lactobacillus johnsonii* may be responsible for the elevated fecal TCDCA levels observed in the HFD group, while *L. reuteri* and *F. rodentium* were positively correlated with body weight gain. Conversely, *Duncaniella freteri* and *D. newyorkensis* were associated with increased LCA and UDCA levels in the HNB group. In contrast, *D. dubosii* and *Anaerobacterium chartisolvens*, which were more abundant in the ND group, appeared to be negatively correlated with DCA levels in the host (Figure 9).

## Discussion

This study aimed to investigate the preventive effects of nobiletin on HFD-induced obesity-related muscle atrophy through bile acid regulation. Our findings demonstrated that nobiletin intervention significantly affected the bile acid synthesis pathway in the liver by upregulating CYP7A1 expression and inhibiting the FGFR/FXR/ERK pathway. Additionally, nobiletin modulated the bile acid composition in the ileum and feces, possibly through modulating the gut microbiota. Furthermore, muscle atrophy-related proteins (e.g., p-4EBP1, TRIM63, and FBXO32) were suppressed, while mTOR/AKT/p70S6K and FOXO3a phosphorylation were enhanced in the nobiletin-supplemented group. Moreover, the FGF15/FGFR4/ERK pathway was activated, potentially due to the reconfiguration of bile acid profiles.

Bile acids not only assist in lipid metabolism in the intestine but also act as signaling molecules involved in lipid, glucose, and energy metabolism pathways. Additionally, they are metabolized by gut microbiota in the intestine through processes such as dehydroxylation, dehydration, epimerization, and amino acid deconjugation, resulting in various bile acid structures with differing activation and inhibition capabilities for the bile acid nuclear receptor FXR.^23^ These bile acids can generally be classified into FXR agonists (e.g., CA, CDCA, TCA, TCDCA) and FXR antagonists (e.g., UDCA, TUDCA, TβMCA, αMCA). In the measurement of bile acids from colonic contents, the results showed a decrease in UDCA levels in the HFD group of mice, while nobiletin intervention led to a significant increase in UDCA levels. According to relevant literature, UDCA has been used to treat noncholestatic and nonhepatobiliary diseases and has been shown to reduce the risk of developing colorectal cancer.^24,25^ In addition, a previous study suggested that UDCA treatment improved muscle contractility in patients by decreasing cholesterol content in muscle.^26^ However, Orozco-Aguilar et al. reported that UDCA administration could lead to decreased protein synthesis, which may consequently contribute to the development of sarcopenia.^27^ Our findings suggest that nobiletin may increase fecal UDCA levels; however, the correlation between elevated fecal UDCA and the prevention of muscle atrophy remains to be clarified.

As previously mentioned, αMCA acts as an FXR antagonist, and this was confirmed by the bile acid measurements in the ileum (Figure 4), where the HNB group exhibited higher levels of αMCA. Consequently, the expression of the FXR protein in the ileum was downregulated in the HNB group, which further led to a reduction in the downstream FGF15 protein expression. In the enterohepatic circulation, FXR in ileal epithelial cells is a nuclear receptor activated by bile acids. It regulates the expression of target genes such as FGF15/19, which circulate through the portal vein to the liver, where they bind to the heterodimeric receptor (FGFR4/βKlotho) on hepatocytes. This binding reduces bile acid synthesis in the liver by inhibiting the transcription of CYP7A1.^28^ In our study, the reduction of the ileal FXR-FGF15 signaling may have activated the hepatic CYP7A1 protein in the HNB group. Relevant studies have indicated that increased CYP7A1 expression can prevent hepatic cholesterol accumulation and hypercholesterolemia induced by a Western diet in mice.^29^ This could be achieved by stimulating the conversion of cholesterol into bile acids in the liver, thereby reducing blood cholesterol levels and alleviating the effects of HFD-induced obesity, insulin resistance, and fatty liver disease. Our findings showed that serum cholesterol was significantly reduced in the HNB group, accompanied by improvements in fasting glucose levels, HOMA-IR, and OGTT results, which were consistent with previous studies. In addition, enhanced skeletal muscle function could facilitate glucose regulation,^30^ while a reduced capacity for glucose disposal has been reported to be positively correlated with loss of muscle mass.^31^ Therefore, the prevention of muscle atrophy and the enhancement of skeletal muscle synthesis might contribute to the improvement in glucose homeostasis observed in our study.

A recent study showed that treatment with FGF19 could significantly increase skeletal muscle fiber size and ameliorate metabolic perturbations in mice.^32^ In our study, the FGF15/FGFR4/ERK signaling pathway was significantly activated in the skeletal muscle tissues of nobiletin-supplemented mice, consistent with previous studies. While the FGF15/FGFR/ERK pathway was inhibited in the liver to promote bile acid synthesis, it was activated in skeletal muscle to prevent muscle atrophy in the HNB group. We speculate that this difference in pathway activation results from the FXR antagonism caused by bile acids in the liver.

In summary, this study investigated the preventive effects of nobiletin on HFD-induced obesity-related muscle atrophy through bile acid regulation. Our findings demonstrated that nobiletin intervention significantly influenced the bile acid synthesis pathway in the liver by upregulating CYP7A1 expression and inhibiting the FGFR/FXR/ERK pathway. Additionally, nobiletin modulated bile acid composition in the ileum and feces, possibly through gut microbiota modulation. Importantly, nobiletin supplementation suppressed muscle atrophy-related proteins and enhanced protein synthesis in skeletal muscle tissues. Furthermore, the FGF15/FGFR4/ERK signaling pathway was significantly activated in skeletal muscle, which contributed to obesity-related sarcopenia prevention and improvement in glucose homeostasis. Overall, these results indicate that nobiletin exerts a protective effect against HFD-induced muscle atrophy, offering a promising approach to address obesity-related complications.

## Data Availability

The data that support the findings of this study are available in the Supporting Information of this article.

## Supporting Information Available

Nobiletin supplementation reverses the detrimental effects of high-fat diet feeding on organ health (Figure S1); ameliorative effect of nobiletin on the liver and gastrocnemius tissues of mice on a high-fat diet (Figure S2); effect of nobiletin on the quadriceps tissues of mice on a high-fat diet (Figure S3); nobiletin could not reverse the development of atrophy markers induced by dexamethasone in differentiated C2C12 cells (Figure S4); and effect of nobiletin on MyoD1 and myogenin expressions in CA- and DCA-treated differentiated C2C12 cells (Figure S5) (PDF)
