# Supplementary material for: Nobiletin Enhances Skeletal Muscle Mass and Modulates Bile Acid Composition in Diet-Induced Obese Mice
Source: J Agric Food Chem. 2025 Apr 7;73(15):9076–87. doi: 10.1021/acs.jafc.5c00255 (PMC12007094; doi:10.1021/acs.jafc.5c00255)
Supplement: Supplementary file 1 — jf5c00255_si_001.pdf [file jf5c00255_si_001.pdf]

Supporting materials

Nobiletin Enhances Skeletal Muscle Mass and Modulates Bile Acid Composition in Diet-Induced Obese Mice

Yen-Chun Koh[†#], Chien-Ping Liu[†#], Siu-Yi Leung[†], Wei-Sheng Lin[†‡], Pin-Yu Ho[†], Chi-Tang Ho[§], Min-Hsiung Pan[†,‖ *]

[†] Institute of Food Sciences and Technology, National Taiwan University, 10617 Taipei, Taiwan

[‡] Department of Food Science, National Quemoy University, 89250 Quemoy, Taiwan

[§] Department of Food Science, Rutgers University, New Brunswick, 08901 New Jersey, USA

[‖] Department of Medical Research, China Medical University Hospital, China Medical University, 40402 Taichung City, Taiwan

# The authors contribute equally

*** Please send all correspondence to:**

Dr. Min-Hsiung Pan

Institute of Food Science and Technology,

National Taiwan University,

No. 1, Section 4, Roosevelt Road, Taipei 10617, Taiwan.

Tel. no. +886-2-33664133

Fax. no. +886-2-33661771

E-mail: mhpan@ntu.edu.tw

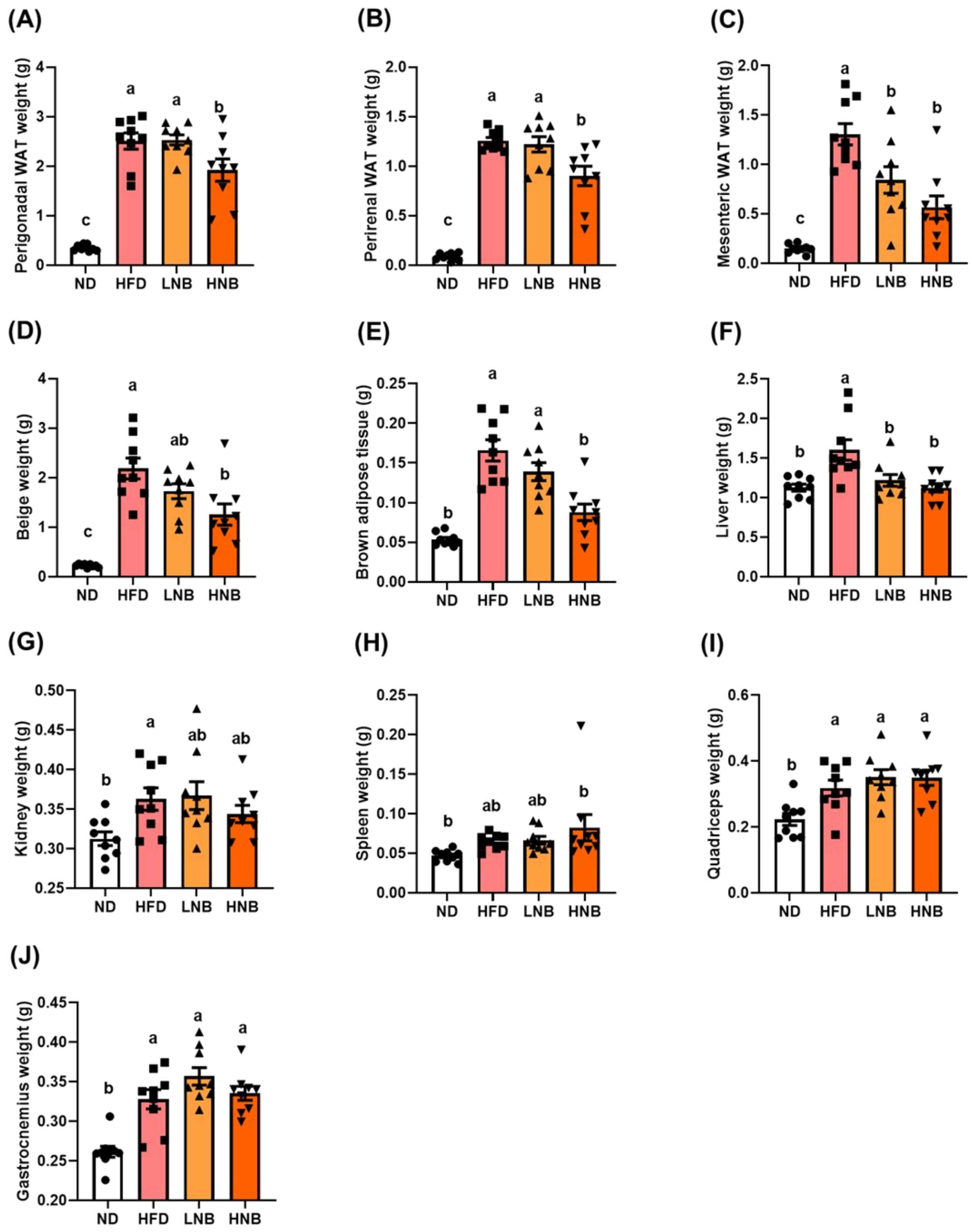

Figure S1. Nobiletin supplementation reverses the detrimental effects of high-fat diet feeding on organ health.

Shown are the organ weight of (A) perigonadal white adipose tissue, (B) perirenal white adipose tissue, (C) mesenteric white adipose tissue, (D) beige adipose tissue, and (E) brown adipose tissue. The weights of (F) liver, (G) kidney, (H) spleen, (I) quadriceps, and (J) gastrocnemius muscle tissues are

also presented. All data are presented as means ± S.E., N = 8. Different lowercase letters indicate significant differences among groups, as determined by ANOVA followed by Tukey's post hoc tests.

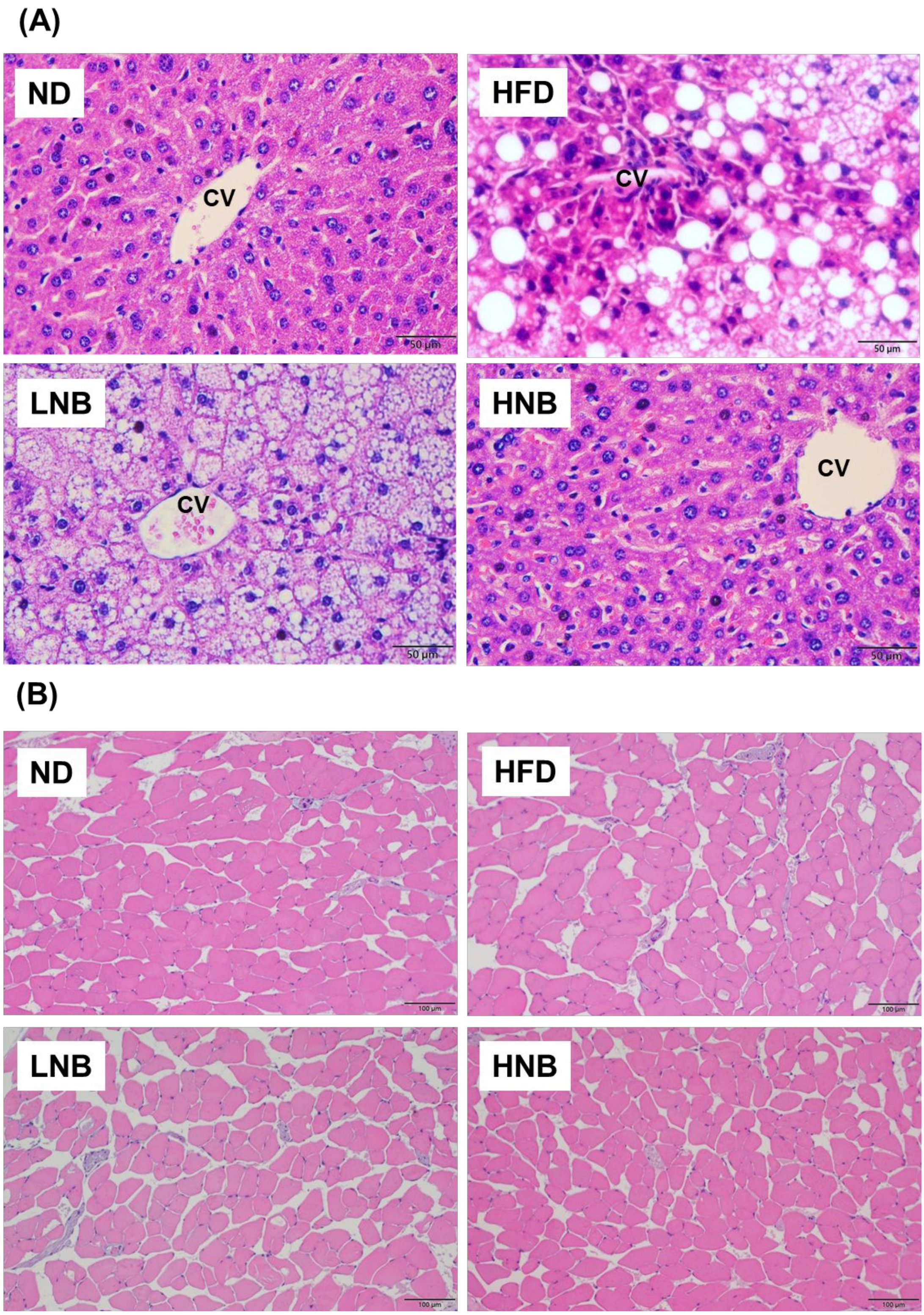

Figure S2. The ameliorative effect of nobiletin on the liver and gastrocnemius tissues of mice on a high-fat diet.

H&E staining of liver and gastrocnemius tissue sections. The images of livers were viewed at 400x magnification (scale bar = 50 μm). The images of

gastrocnemius tissues were viewed at 200x magnification (scale bar = 100 μm).

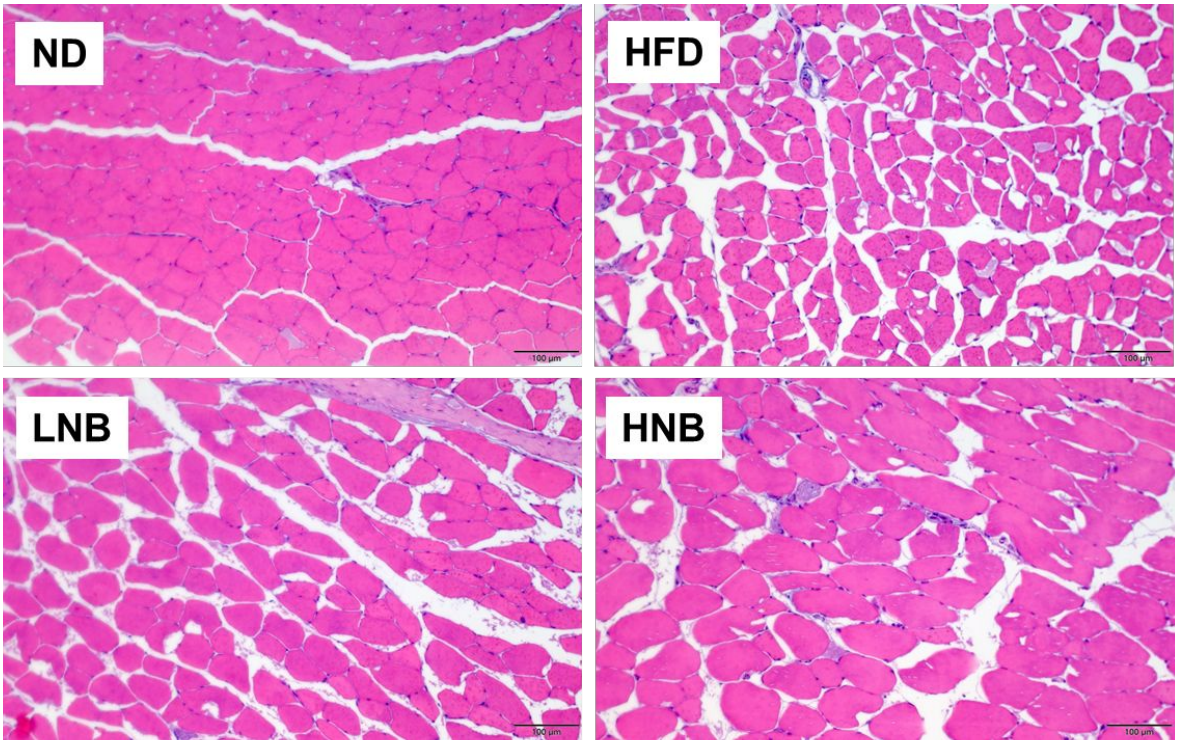

Figure S3. The effect of nobiletin on the quadriceps tissues of mice on a high-fat diet.

H&E staining of quadriceps tissue sections. The images of quadriceps tissues were viewed at 200x magnification (scale bar = 100 μm).

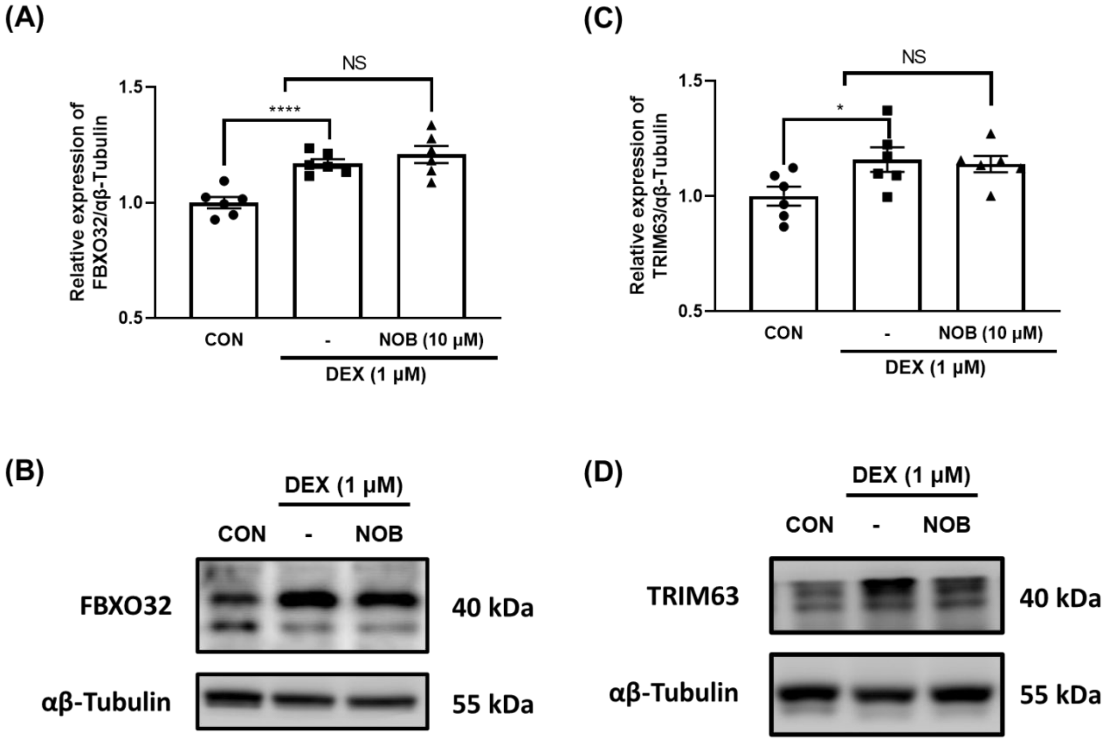

Figure S4. Nobiletin could not reverse the development of atrophy markers induced by dexamethasone in differentiated C2C12 cells.

(A) Quantification and (B) representative western blot images showing the expression of FbXO32 in DEX-induced myotubes after 24-hour intervention with NOB. (C) Quantification and (D) representative western blot images showing the expression of FbXO32 in DEX-induced myotubes after 24-hour intervention with NOB. The symbols (*) and (****) indicate significant differences compared to the induced group, with *p*-values less than 0.05 and 0.001, respectively, determined by Student's t-test.

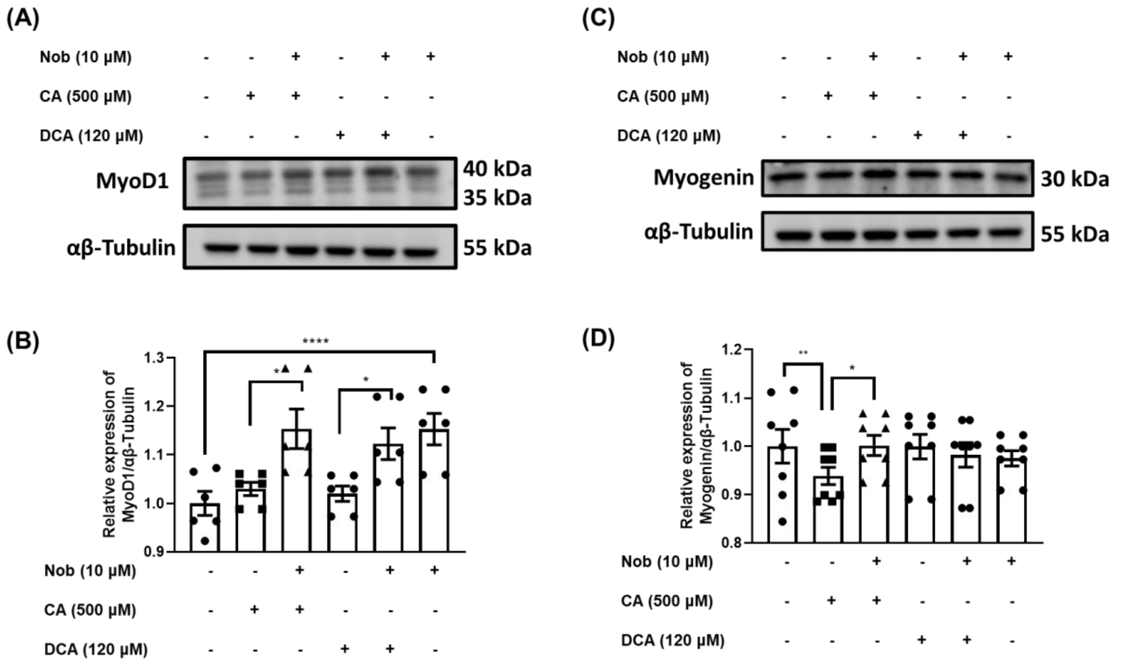

Figure S5. The effect of nobiletin on MyoD1 and Myogenin expressions in CA and DCA-treated differentiated C2C12 cells.

(A) Quantification and (B) representative western blot images showing the expression of MyoD1 in CA and DCA-induced myotubes after 24-hour intervention with NOB. (C) Quantification and (D) representative western blot images showing the expression of Myogenin in CA and DCA-induced myotubes after 24-hour intervention with NOB. The symbols (*), (**) and (****) indicate significant differences compared to the induced group, with *p*-values less than 0.05, 0.01 and 0.001, respectively, determined by Student's t-test.
